# Cardiac Magnetic Resonance in heart transplant patients: diagnostic value of quantitative tissue markers (T2 mapping and ECV) for acute cardiac rejection diagnosis

**DOI:** 10.1186/1532-429X-18-S1-O47

**Published:** 2016-01-27

**Authors:** Emmanuelle Vermes, Clemence Pantaleon, Julien pucheux, Michel Aupart, Nicolas Cazeneuve, Laurent Brunereau

**Affiliations:** 1 Cardiac imaging, CHU Trousseau, Tours, France; 2 Heart surgery, CHU Trousseau, Tours, France

## Background

The diagnosis of acute cardiac rejection (AR) still requires invasive technique with endomyocardial biopsy (EMB) which has important risks and limitations.

Cardiac Magnetic Resonance (CMR) with recently T2 and T1 mapping is a promising technique for characterizing myocardial tissue.

The purpose of the study was to evaluate T2, T1 and extracellular volume (ECV) quantification as novel tissue markers to diagnose acute cardiac rejection.

## Methods

CMR was prospectively performed in heart transplant patients within 6 ± 13 days of routine EMB and before acute rejection therapy. Images were acquired on a 1.5 Tesla scanner including T2 mapping (T2 prepared-SSFP) and T1 mapping using a modified look locker inversion recovery sequence (MOLLI) at basal, mid and apical level in short axis view.

T2 and T1 values were measured before and 15 minutes (for T1 mapping) after contrast administration. The results are expressed by the median and the 5^th^ and the 95^th^ percentiles.

## Results

Twenty five patients (age 56 ± 14 years) were included providing 38 comparisons CMR/EMB. Acute rejection (cellular, humoral or clinical symptoms) was diagnosed in 11 patients.

Patients with AR had significantly higher global T2 values at 3 levels (59 ms [54-63] vs 51 ms [49-55], P = 0.0025 at basal; 57 ms [55-65] vs 52 ms [50-54], P = 0.001 at median and 60 ms [54-66] vs 55 ms 51-57], P = 0.013 at apical level). The area under the curve (AUC) for each level was: 0.83; 0.58 and 0.77 respectively.

Patients with AR had significantly higher ECV at basal and median level: 35% [33-39] vs 27% [25-31] P = 0.003 and 33% [28-39] vs 27%[24-31], P = 0.025 respectively. The AUC for each level was; 0.85 and 0.75 respectively.

The sensitivity, specificity and diagnosis accuracy for basal T2 (cutoff : 57.7 ms) were 70%, 96% and 79% respectively; basal ECV: (cutoff 31.2%) 89%, 77% and 81% respectively. The best AUC (0.88) was obtained when we combined basal T2 and basal ECV.

## Conclusions

In heart transplant patients, a combined CMR approach using T2 mapping and ECV quantification provides a high diagnostic accuracy for acute rejection diagnosis and could potentially decrease the number of routine EMB. Further studies are required to confirm these data.

